# Bellevue Hospital Medical College

**Published:** 1870-12

**Authors:** 


### Editorial.

### Bellevue Hospital Medical College.

We notice in the *New York Medical Journal* for December, the following invitation extended to Prof. James P. White, M. D., of Buffalo, by the Faculty of the Bellevue Hospital Medical College. It will be understood, that Prof. George T. Elliott, being incapacitated by illness from delivering the lectures during the present session, the course has been given by Prof. White, and that this invitation is in expression of the appreciation of his services:

BELLEVUE HOSPITAL MEDICAL COLLEGE,
FOOT OF TWENTY-SIXTH STREET, EAST RIVER,
NEW YORK, November 9, 1870.

PROF. JAMES P. WHITE—

My Dear Sir: I have been directed to respectfully request you to name a day when it will be agreeable for you to meet our Faculty at dinner. We are anxious to have an opportunity of meeting you socially, as a Faculty, to express our appreciation of your noble and generous act, which has given us the advantage of your large professional experience and wide reputation, in our course on Obstetrics. With sentiments of the highest esteem and respect,

I remain your obedient servant,

A. FLINT, JR., Secretary."

